# Critical Issues of Working during the COVID-19 Pandemic: Italian Healthcare Workers’ Experience

**DOI:** 10.3390/ijerph19020927

**Published:** 2022-01-14

**Authors:** Silvia Vicentini, Arianna Mercurio, Carolina Romascu, Martina Battaglia, Noemi Tribbia, Francesca Siviero, Antonello Grossi, Maria Maddalena Martucci, Diego De Leo

**Affiliations:** 1 Department of Mental Health, Local Health Unit 5, 45100 Rovigo, Italy; dott.silviavicentini@gmail.com (S.V.); arianna.mercurio@yahoo.it (A.M.); carolina.romascu96@gmail.com (C.R.); martina.battaglia.3@studenti.unipd.it (M.B.); noemi.tribbia@outlook.it (N.T.); siviero.francesca@gmail.com (F.S.); antonello.grossi@aulss5.veneto.it (A.G.); 2 Australian Institute for Suicide Research and Prevention, Griffith University, Brisbane 4122, Australia

**Keywords:** COVID-19, online survey, healthcare workers, mental health, Italy

## Abstract

*Background*: The COVID-19 pandemic has strongly impacted on healthcare services’ organization and healthcare workers’ mental health, increasing the risk of psychological symptoms and burnout. Italy has been one of the most affected countries, especially the northern regions, even with exceptions in some rural provinces. *Aim*: We chose to investigate the mental health conditions of healthcare workers operating in the rural province of Rovigo (a small town in Veneto, northern Italy), where relatively few deaths and contagions were reported during the pandemic, even if Veneto—globally—was one of the most affected regions of Italy. We wanted to verify the psychological outcomes of health workers operating in a context where the impact of the pandemic appeared to be relatively mild. *Methods*: Through an online survey, we investigated perceived difficulties at work and in daily life, perceived loneliness and social support, coping strategies, and level of psychological distress (sample size: 749; mean age = 48.04 years, SD = 10.66). The questionnaire had both open- (2) and close-ended questions (5 single-choice and 13 multiple-choice). We verified possible associations between sex, age group, work department and percentage of responses with chi-square tests of independence on each question. Data cleaning excluded all contradictory answers from the multiple-choice questions from the analyses (final sample size: 640). *Results*: Frontliners and non-frontliners reported a similar experience of the COVID-19 pandemic (without significant differences in perceived difficulties, coping strategies and sources of support). Nevertheless, they still reported various forms of negative emotions (e.g., helplessness—40.94%; sadness—36.56%; frustration—32.66%) and lack of support from the health organization (especially frontliners—28.72%). However, psychological help was scarcely requested. *Conclusions*: Despite the province not being massively affected by the pandemic, healthcare workers felt the need for clearer and more supportive guidance. They seem to perceive collective opportunities to share needs and difficulties as more useful than individual interventions (as those provided by the ad hoc created listening service).

## 1. Introduction

### 1.1. The Spread of COVID-19 in the Italian Context

In Italy, the pandemic followed a three-phase trend. The First Wave (February–May 2020) was characterized by a quick spread of cases and deaths; it was followed by a reduction in the number of cases and deaths in the Transition Phase (June–September 2020). The Second Wave started at the end of September 2020; it showed a rapid increase in the number of cases, and a decline in December 2020 [1]. Then, COVID-19′s spread increased again at the beginning of 2021, with a strong impact on mortality (especially in the northern regions: +75% of excess mortality just in January 2021), which characterized the second part of the Second Wave (January–April 2021) [1,2].

The government imposed various lockdown measures and restrictions to contain the spread of the virus. Due to the decentralized organization of the Italian healthcare system, different types of protocols were applied locally to contain the pandemic; as a consequence, nearby regions showed heterogeneous outcomes. For instance, the Veneto region, initially struck by a severe spread of the virus, chose to adopt a multi-pronged strategy of containment [3]. This, at the end of the First Wave, led to a better outcome in terms of excess mortality (+19.4%) than the nearby Lombardy (+118.8% of excess mortality—the highest in Italy). Then, between October and December 2020, Veneto’s excess mortality increased by +44.4%, exceeding the First Wave figures [2]. This trend was confirmed in other Northern regions—such as Aosta Valley (+63.7% vs. a previous +42.6%), Piedmont (+53% vs. +47.5%), Friuli-Venezia Giulia (+45.6% vs. +9%) and Emilia-Romagna (+43.6% vs. +25.4%). The latter area, together with Veneto and Lombardy, explained 50% of the total excess mortality in January 2021 [2]. It must be said that COVID-19 incidence was significantly lower in Southern Italy, even in the first months of 2021 (especially in the most rural provinces of Sardinia, Calabria and Sicily) [1,2].

### 1.2. How Healthcare Services Coped with the Pandemic

Both the general population and medical staff developed a general sense of uncertainty and fear for the future [4,5,6]. In Italy, like elsewhere, the pandemic caused loneliness and fear of death, while also causing economic and social disruption [7,8]. Healthcare services had to face unprecedented pressure and sustain a lot of organizational changes. This was common across Europe [9].

Due to exposure to the contagion, health care workers (HCWs) were a high-risk population for physical and psychological distress. In a few months, they experienced a distressing increase in workload along with a lack of adequate personal protective equipment (PPE) in terms of quality, quantity, and training [10,11,12,13]. The general unpreparedness of the healthcare services was an important source of distress for HCWs [14]. In addition, they felt lonely [6,7,15,16], and had concerns about the danger of being infected and infecting others [4,10,12,16,17,18,19,20]. Burnout was frequently reported [19,21,22].

As confirmed by Serrano-Ripoll et al. [23], in times of epidemic outbreaks, the risk of developing psychological distress significantly increases for HCWs. Several reviews [21,24,25] and cross-sectional studies on the COVID-19 pandemic [15,16,18,26,27] have shown that anxiety, depression, sleep disturbances, and high levels of stress (and sometimes post-traumatic stress disorder) are mental health problems (MHPs) that are frequently experienced among HCWs in different countries, including Italy [19,20,28].

In Italy—as reported by Costantini et al. [10] and Felice et al. [11]—various measures to counteract the pandemic were adopted; for example, procedures for the management and notification of COVID-19 cases, implementation of dedicated intensive care units (ICUs), and redistribution of roles and tasks beyond one’s own medical specialization. In addition, changes in admission criteria and practices, and an absence of specific guidance and preparation in the care of COVID-19 patients were ubiquitous. Felice et al. [11] highlighted that only 3.3% of interviewed HCWs were receiving psychological support, despite 63.7% of them believing that this would be needed.

### 1.3. Risk and Protective Factors

The Asian and European literature showed that being female and young and working as a frontliner are generally associated with more distress and related MHPs [15,21,25,26]. This has been confirmed even in the Italian context [19,21,25,28]. Psychophysical discomfort (e.g., fatigue [7,29]) and various kinds of negative emotions (such as restlessness, helplessness, sadness, frustration, etc.) have been often identified in those HCWs who faced the COVID-19 pandemic worldwide [5,7,14,18,29], even in Italy [6,7,19].

Some studies showed that nurses experienced more symptoms of MHPs than doctors (e.g., [15,21]), while others found the contrary [27], with doctors more vulnerable to burnout than nurses [19]. In China, Zhang et al. [27] found the frontliners that they recruited did not perceive severe MHPs.

These results suggest that environmental variables could protect HCWs’ mental health. Indeed, research in Asian and European contexts [4,17,29,30]—including Italy [12]—highlighted that positive emotions from the work environment, perceived social support (from colleagues, friends and/or family), use of adequate PPE (with appropriate training for utilization), clear guidelines and protocols, adoption of proper self-care coping and management styles, and good management by the organization could all act as protective factors from MHPs.

### 1.4. The Survey

In this critical scenario, the Mental Health Department of the Azienda ULSS-5 Polesana (AUP) decided to investigate the impact of the COVID-19 pandemic on the HCWs employed in the healthcare services of the Rovigo province (a small town in Veneto, northern Italy). The aim of the survey was to investigate the specific COVID-19 experiences of HCWs operating in a public setting, thus informing the leaders of the organization of possible remedies to critical issues. We chose this area because it represents a peculiar case: despite Veneto being one of the regions most affected by COVID-19 [1,2,3], the Rovigo province always recorded relatively fewer deaths and contagions than the rest of the region [31]. So, we wanted to probe if we could really expect relatively better psychological outcomes in HCWs of this particular area.

## 2. Materials and Methods

### 2.1. Study Design and Setting

In the context of a large project on the prevention of depression and promotion of public health, we administrated an online survey about the personal and professional experiences of the HCWs employed in the AUP during the second part of the Second Wave of the COVID-19 pandemic. The study was designed to be an observational and cross-sectional survey. Our aims were to capture: (1) the perceived psychological impact of the pandemic on HCWs of the Rovigo province and (2) the most reported risks and protective factors.

The AUP General Direction formally approved the questionnaire and authorized the investigation, which implied that all the requirements to perform the research had been fulfilled (approval number: no. 0116438/VII.5). The latter was widely publicized, and the questionnaire was disseminated via corporate emails and on posters with a QR code. It was also hung up and displayed with information on the billboards of common areas of all healthcare services. The survey was administrated between 22 December 2020 and 21 March 2021. The inclusion criteria were: (a) being an HCW; (b) working at the healthcare services of the AUP. All participants were recruited online and anonymously.

### 2.2. Instruments

We adopted an online questionnaire entitled “The impact of the pandemic in the life stories of the healthcare workers”, specifically designed for this survey. It had a main quantitative part (with 18 close-ended questions, focus of this paper), and a facultative qualitative part (with two open-ended questions, not analyzed here). The quantitative part was composed of five single-choice, close-ended questions and thirteen multiple-choice, close-ended ones.

The five single-choice questions inquired: (a) perceived risk of contagion (1); (b) time spent in isolation (1); (c) how the HCWs worked during the pandemic (from home or in person) (1); (d) impact of pandemic on household income (1); and (e) perceived reduction in income (1).

The thirteen multiple-choice questions inquired: (f) difficulties experienced during the pandemic (1); (g) coping strategies (1); (h) perceived social support (1); (i) loneliness (1); (l) quality of life (3); (m) positive and negative impact of restrictions (2); (n) perceived consequences at work (2); and (o) perceived distress and negative emotions (2).

Demographic data were collected.

### 2.3. Data Analysis

The responses to the questionnaire were analyzed through the Python programming language [32]. Pearson’s chi-square tests were performed on sex, age groups and work department to verify the representativeness of the sample in terms of HCWs employed in the AUP. Chi-square tests of independence were performed on the single-choice questions to verify if there were associations between sex, age group, work department and percentage of responses. For these answers, we considered the data derived from the original sample.

The multiple-choice questions were analyzed capturing the mode and identifying the major frequencies in the responses chosen by 25% or more of participants, after data cleaning to exclude contradictory data. Chi-square tests were performed on each response separately to verify if there were associations between sex, work department and age group and each most chosen option. Yates’ continuity correction was applied on every chi-square that had one degree of freedom.

## 3. Results

### 3.1. Descriptive Data

Among the 3084 HCWs employed in the AUP, 749 (24.2%) individuals (mean age = 48.04 years; SD = 10.66) agreed to participate in the study and answered the questionnaire.

Data cleaning was performed on multiple-choice questions to exclude those participants who marked both “Yes” and “No” statements to the same question. The purpose was to avoid the inclusion of contradictory data. After this cleaning, the sample was reduced to 640 participants. The five single-choice questions were not influenced by the data cleaning because, due to their nature, they were not vulnerable to contradictory answers.

Sample characteristics are reported in Table 1. At the time of the online publication of the questionnaire, only 12 HCWs (1.88%) completed the questionnaire when the first dose of the COVID-19 vaccine was not available, and 628 (98.13%) while it was available.

Pearson’s chi-square test was performed considering sex (with Yates’ continuity correction), age group and work department (excluding “others” and “not fully employed”) to compare the sample distribution with the population one. The results of the chi-square tests were significant for the categories sex (*X*^2^(1, *N* = 749) = 1,7; *p* = 0.019), age group (*X*^2^(4, *N* = 749) = 7,7; *p* = 0.01) and work department (*X*^2^(3, *N* = 724) = 44,95; *p* < 0.001).

### 3.2. Main Results: Single-Choice Questions

The most frequent answers showed that 533 participants (71.16%) had “never” been quarantined and 92% (*n* = 689) of them declared they used to go to work “in person, every day”. Overall, they affirmed their family did not experience huge economic concerns (*n* = 364–48.6%), but 320 participants (42.72%) perceived a reduction of 15% in their income, while 259 (35.58%) affirmed their income did not diminish. Specifically, a chi-square test of independence showed that this difference was significant between males and females (*X*^2^(6, *N* = 747) = 20,75, *p* = 0.002). Most males (*n* = 77–44.25%) responded that they did not perceive a reduction in income, while most females (*n* = 262–45.55%) perceived a reduction of 15%.

Considering the risk of infection, most participants (*n* = 293–39.12%) perceived a “light risk” of being infected at work, but the chi-square test of independence showed a significant association between the perceived risk and work department (*X*^2^(12, *N* = 724) = 107,40, *p* < 0.001). Thus, most frontliners (*n* = 44–39.29%) perceived a “very high risk” of infection, most administration workers (*n* = 33–33.98%) a “small risk”, and most community workers (*n* = 99–45.41%) and general hospital workers (*n* = 123–42.19%) a “light risk”.

The mode trend of these five questions did not change considering age groups.

### 3.3. Main Results: Multiple-Choice Questions

#### 3.3.1. Negative Psychological Outcomes

For HCWs, the main difficulties of the pandemic were the “Fear that the people I care about will get sick” (*n* = 419–65.47%), “The feeling of uncertainty” (*n* = 257–40.16%) and “Worries of being infected” (*n* = 173–27.03%). Regarding their experience of loneliness, they declared that it changed during the pandemic because “It is worse when it is imposed by others” (*n* = 261–40.78%). Many people said that the situation worsened since the pandemic began because “In a prolonged state of emergency, things always get worse” (*n* = 306–47.81%), “The burden of fatigue increases” (*n* = 198–30.94%) and “The belief of being prepared makes you underestimate the situation” (*n* = 182–28.44%).

They reported that their psychophysical distress increased mostly due to “The heavy workload” (*n* = 192–30%) and “The self-isolation and the changes in my life-style” (*n* = 178–27.81%), and many HCWs declared they have experienced negative emotions such as “Helplessness” (*n* = 262–40.94%) “Sadness” (*n* = 234–36.56%); “Fear” (*n* = 212–33.13%); “Frustration” (*n* = 209–32.66%); and “Restlessness” (*n* = 171–26.72%). Regarding work conditions, most HCWs (*n* = 412–64.38%) stated that they did not change work area, while 53.91% (*n* = 345) declared that they did not feel adequately trained to deal with the emergency (“Despite my long professional experience, I was not prepared to face such a severe condition”).

#### 3.3.2. Positive Psychological Outcomes

Most HCWs coped with the difficulties mostly by “Doing the things I like the most and I can do under restrictions” (*n* = 342–53.44%), “Keeping myself informed” (*n* = 320–50%) and “Discussing with family and friends” (*n* = 282–44.06%). They also declared that they were able to ask for help to “family” members (*n* = 223–34.84%) and “colleagues” (*n* = 158–25%), but 237 of them (37.03%) stated they did not ask anyone for help. Overall, HCWs declared that the pandemic influenced their quality of life, making them realize “how many things are neglected in the name of the duty of doing” (*n* = 241–37.66%), “how important are the relationships with family and friends” (*n* = 195–30.47%), “how much I spend my time doing what I must do instead of what I would like to do” (*n* = 169–26.41%) and “how much fragile we are” (*n* = 161–25.16%). Moreover, most of them stated that “Keeping in touch from a distance with people” (*n* = 401–62.66%), “Doing things I like to do” (*n* = 306–47.81%) and “Spending my time in family” (*n* = 266–41.56%) helped them during lockdowns and periods of restrictions.

Then, they saw benefits in lockdown measures such as “Realizing that we are all vulnerable” (*n* = 244–38.13%) and “Giving more value to life” (*n* = 235–36.72%). HCWs also declared that their pandemic experience was not influenced by the place where they live (*n* = 235–36.72%), even if 34.69% of them (*n* = 222) reported that it “was positively influenced by the various services near me”.

For an overview of main results, see Table 2.

## 4. Discussion

The present study offers a view on the physical and psychological consequences of the COVID-19 pandemic on a sample of HCWs operating in Veneto during the second part of the Second Wave of the pandemic. The day the questionnaire went online (22 December 2020), Veneto was the region with the most new cases (3082) and deaths (150) [33], but in the Rovigo province, the AUP registered less pressure than the other provinces of Veneto. Indeed, despite the heavy national and regional pressure on hospitals, the hospitalized patients for COVID-19 were, respectively 130 when the survey began [34] and 95 when it ended on 21 March 2021 [35], mostly hospitalized in Trecenta (the only COVID hospital of the province).

As seen in previous studies, the HCWs experienced various difficulties during the pandemic, such as feelings of uncertainty (40.16%) [5,8,10] and fear of being infected (27.03%) [10,12,18] or that the people they care about would become ill (65.47%), a worry that could be related to the fear of infecting their family [4,10,12,16,17,20]. Then, feeling unprepared in facing the COVID-19 pandemic’s severity emerged, especially in females (57.11%).

However, our results highlight that, overall, HCWs seemed able to find support in their social network: family (34.84%), colleagues (especially females: 28.25%) and friends (mostly the youngest HCWs: 20–29 years: 44.74%). They were also able to adopt useful self-care coping strategies to deal with the pandemic, such as doing things that they like most (53.44%), keeping themselves informed (especially the oldest HCWs: 50–59 years: 54.41%; 60–70 years: 58.54%) and discussing with family and friends (especially females: 53.61%). These aspects might protect them against mental health problems [17,25,29]. Coherently, in terms of protective factors, some HCWs declared that they rediscovered the importance of relationships with family and friends and the value of life. These factors seemed to increase their perceived quality of life and might have helped them: (a) to see the pandemic as a time of change and growth and to cope better with the psychological distress brought by it [8]; (b) to perceive some competence in dealing with it (e.g., related to their long professional experience: reported by 25% of males).

More than 30% of HCWs declared they did not ask for help (mostly males—50.97%—and the oldest HCWs—e.g., 60–70 years: 53.66%). This might be because the virus did not affect the Rovigo province so massively at the time of the survey; so, in general, HCWs might have perceived a low level of need for support, especially professional support. Indeed, only 15 people (2.34%) affirmed that they asked for psychological help, and only 6 persons utilized the psychological support service created ad hoc by the AUP. In addition, we could not detect significant differences between frontliners and non-frontliners. Most frontliners (45.74%) reported that they were distressed by the heavy workload [17,26], whilst most non-frontliners (28.68%) did not perceive an increase in the level of distress since the beginning of the pandemic. However, they both reported negative emotions such as helplessness, restlessness, sadness, frustration and fear [5,6,7,14,18,19,29].

As already said, very soon the AUP provided a dedicated psychological support service to counteract the emotional impact of the pandemic on its workers. As it turned out, and as seen before, this was scarcely used. This may suggest that HCWs could perceive other forms of interventions as more useful. Collegial interventions such as focus groups between employees and supervisors could be an alternative: they would offer the opportunity to express reciprocal needs and to discuss the problematic aspects of working during the COVID-19 pandemic, with the aim of finding solutions. As a matter of fact, more than 25% of males and frontliners felt prepared enough to deal with the pandemic, but not sufficiently supported by the organization. This lack of perceived organizational support (risk factor for burnout during the pandemic: 22) may be related to a certain level of dissatisfaction with the health organization’s bad COVID-19 management.

## 5. Conclusions

The aim of this survey was to investigate the personal and professional experiences of the COVID-19 pandemic of HCWs from the public health sector, focusing on their perceived risk and protective factors, to obtain useful information about critical issues and to help the leaders of the organization in setting up possible remedies.

As an observational survey, our results offer a contribution to the literature describing the mental health condition of the HCWs operating in a province of northern Italy that was not among the most impacted areas. To our knowledge, surveys of the type reported here are still uncommon from Italy.

The main findings show that the HCWs seemed able: (a) to adopt useful self-care coping strategies to deal with the pandemic and to recognize even some benefits to their quality of life in this situation; (b) to find support in their social network (even if more than 30% declared they did not do this). However, a number of HCWs reported psychological discomfort towards the lack of sufficient organizational support, and negative emotions. These reactions could increase the risk of MHPs and burnout.

The relatively modest spread of the virus in the Rovigo province could explain the relatively similar experience of the pandemic between frontliners and non-frontliners; however, it still was a stressful experience for both, as suggested by the high percentage of negative emotions reported. This suggests that clearer and more supportive guidance would be essential for HCWs in any case. The health organization (AUP) should be able to improve its management strategies of emergencies, and provide adequate protocols and measures of support to prevent distress and the onset of MHPs in HCWs.

## Figures and Tables

**Table 1:** Original (*N* = 749) and clean (*N* = 640) sample characteristics.

Characteristic	Original Sample	Clean Sample
Sex		
Females	575 (76.8%)	485 (75.78%)
Males	174 (23.2%)	155 (24.22%)
Age		
20–29	51 (6.81%) (M = 26.24; SD = 2.21)	38 (5.94%) (M = 26; SD = 2.21)
30–39	118 (15.75%) (M = 38.87; SD = 2.74)	109 (17.03%) (M = 34.83; SD = 2.76)
40–49	169 (22.56%) (M = 44.98; SD = 2.95)	150 (23.44%) (M = 44.98; SD = 2.98)
50–59	311 (41.52%) (M = 54.56; SD = 2.74)	261 (40.78%) (M = 54.46; SD = 2.69)
60–70	100 (13.35%) (M = 62.3; SD = 2.05)	82 (12.81%) (M = 62.33; SD = 2.08)
Job position		
Hospital workers	291 (38.85%)	257 (40.16%)
Community workers	218 (29.12%)	177 (27.66%)
Frontliners	112 (14.95%)	94 (14.69%)
Administration workers	103 (13.75%)	89 (13.91%)
Others	19 (2.54%)	18 (2.81%)
Not fully employed	6 (0.80%)	5 (0.78%)
Educational level		
PhD	8 (1.1%)	7 (1.09%)
Post lauream master	86 (11.35%)	75 (11.72%)
University	393 (52.45%)	344 (53.75%)
High School	221 (29.51%)	185 (28.91%)
Middle School	37 (5%)	25 (3.91%)
Children		
Yes	495 (66.09%)	416 (65%)
No	254 (33.91%)	224 (35%)
Social status		
Married	434 (58%)	377 (58.91%)
In a relationship	124 (16.55%)	102 (15.94%)
Single	100 (13.35%)	89 (13.91%)
Divorced	76 (10.15%)	62 (9.69%)

**Table 2:** Absolute frequencies (No.) and percentages (%) of significant chi-square associations based on clean data.

Question	Option	Categories	No. (%)	*X*^2^ (*df*, *N*)	*p*
2. How do you cope with the pandemic difficulties?	Discussing with family and friends	Females	233 (48.04%)	7.77 (1, 1110)	=0.005
		Males	49 (31.61%)		
3. Did you have the opportunity to ask for help for the pandemic difficulties?	No, I did not ask for help	Females	158 (32.58%)	25.34 (1, 894)	<0.001
		Males	79 (50.97%)		
	Yes, I did from colleagues	Females	137 (28.25%)	7.22 (1, 894)	=0.007
		Males	21 (13.55%)		
11. Did you feel professionally prepared to deal with the pandemic emergency?	Yes, I did because I know how to intervene in critical situations because of my long professional experience	Females	54 (11.13%)	12.93 (1, 737)	<0.001
		Males	38 (25%)		
	Despite my long professional experience, I was not prepared to face a such severe condition	Females	277 (57.11%)	11.80 (1, 737)	<0.001
		Males	68 (43.87%)		
	I felt prepared, but not supported by the organization	Females	8 (12%)	9.7 (1, 737)	=0.002
		Males	38 (25%)		
10. Did you change working area during the pandemic?	No, I didn’t	Non frontliners	352 (67.30%)	19.60 (1, 652)	<0.001
		Frontliners	46 (48.94%)		
11. Did you feel professionally prepared to deal with the pandemic emergency?	I felt prepared, but not supported by the organization	Non frontliners	65 (12.43%)	10.08 (1, 652)	=0.001
		Frontliners	27 (28.72%)		
12. Did you perceive an increased psychophysical distress?	No, I didn’t	Non frontliners	150 (26.68%)	7.46 (1, 1008)	=0.006
		Frontliners	16 (17.02%)		
	Yes, I did because of the heavy workload	Non frontliners	141 (27%)	5.37 (1, 1008)	=0.02
		Frontliners	43 (45.74%)		
2. How do you cope with the pandemic difficulties?	Keeping myself informed	20–29 y	13 (34.21%)	11 (4, 1110)	=0.03
		30–39 y	42 (38.53%)		
		40–49 y	75 (50%)		
		50–59 y	142 (54.41%)		
		60–70 y	48 (58.54%)		
3. Did you have the opportunity to ask for help for the pandemic difficulties?	No, I did not ask for help	20–29 y	4 (10.53%)	36.77 (4, 894)	<0.001
		30–39 y	36 (33.03%)		
		40–49 y	50 (33.33%)		
		50–59 y	103 (39.46%)		
		60–70 y	44 (53.66%)		
	Yes, I did from friends	20–29 y	17 (44.74%)	23.68 (4, 894)	<0.001
		30–39 y	28 (25.69%)		
		40–49 y	36 (24%)		
		50–59 y	35 (13.41%)		
		60–70 y	7 (8.54%)		
7. What did you perceive relevant for your personal wellbeing during restrictions?	Spending my time in family	20–29 y	7 (18.42%)	9.99 (4, 1382)	=0.04
		30–39 y	53 (48.62%)		
		40–49 y	75 (50%)		
		50–59 y	105 (44.44%)		
		60–70 y	26 (31.70%)		
11. Did you feel professionally prepared to deal with the pandemic emergency?	No, I didn’t because it’s my first professional experience	20–29 y	13 (34.21%)	54.45 (4, 737)	<0.001
		30–39 y	6 (5.50%)		
		40–49 y	6 (4%)		
		50–59 y	10 (3.83%)		
		60–70 y	2 (2.44%)		
12. Did you perceive an increased psychophysical distress?	No, I don’t	20–29 y	3 (7.89%)	44.54 (1, 1054)	<0.001
		30–39 y	18 (16.51%)		
		40–49 y	32 (21.33%)		
		50–59 y	86 (32.95%)		
		60–70 y	31 (37.80%)		

The clean sample size is 640 for the comparison by sex and age (see Table 1 for details) and 617 for the comparison by work department. “Frontliners” are those who work in COVID-19 wards (*n* = 94). “Non frontliners” are administration, community and hospital workers (*n* = 523).

## Data Availability

The data presented in this study are available on request from the corresponding author. The data are not publicly available due to data sensibility.
